# Oral nutrition or water loading before hip replacement surgery; a randomized clinical trial

**DOI:** 10.1186/1745-6215-13-97

**Published:** 2012-07-02

**Authors:** Stefan Ljunggren, Robert G Hahn

**Affiliations:** 1 Research Unit, Södertälje Hospital, House 18, 581 85, Södertälje, Sweden; 2 Department of Clinical Science and Education, Karolinska Institutet, Södersjukhuset, Stockholm, Sweden; 3 Section for Anesthesia, Faculty of Health Sciences, Linköping University, Linköping, Sweden

**Keywords:** Glucose tolerance test, Glucose clearance, Insulin resistance, Cortisol, complications, W-BQ12, Health index

## Abstract

**Background:**

Surgery induces insulin resistance that might be alleviated by a nutritional drink given preoperatively. The authors hypothesized that some of the beneficial effects of the drink could be attributed to the volume component (approximately 1 L) rather than to the nutrients.

**Methods:**

Sixty patients scheduled for elective total hip replacement under spinal anesthesia were recruited to a clinical trial, and randomly allocated to preoperative fasting, to oral ingestion of tap water, or to oral ingestion of a carbohydrate drink. An intravenous glucose tolerance test calculated glucose clearance and insulin sensitivity on the day before surgery, in the postoperative ward, and on the day after surgery. Other parameters were stress (cortisol in plasma and urine), muscle catabolism (urinary 3-methylhistidine), and wellbeing.

**Results:**

Fifty-seven patients completed the study. In the postoperative ward, the glucose clearance and the insulin response had decreased from the previous day by 23% and 36%, respectively. Insulin sensitivity did not decrease until the next morning (−48%) and was due to an increased insulin response (+51%). Cortisol excretion was highest on the day of surgery, while 3-methylhistidine increased 1 day later. Follow-up on the third postoperative day showed an average of 1.5 complications per patient. Wellbeing was better 2 weeks after than before the surgery. None of the measured parameters differed significantly between the study groups.

**Conclusions:**

Preoperative ingestion of tap water or a nutritional drink had no statistically significant effect on glucose clearance, insulin sensitivity, postoperative complications, or wellbeing in patients undergoing elective hip surgery.

**Trial registration:**

Registration number: NCT 01211184 (http://www.clinicaltrials.gov)

## Background

Elective hip replacement, which is a very common operation performed worldwide, involves a convalescence period of many weeks [1]. The surgery creates a cascade of reactions in the body, including insulin resistance [2], stress hormone secretion, and, possibly, an imbalance in body fluid composition. These effects, in turn, create physical reactions, including degradation of muscle [3,4].

Postoperative insulin resistance has been linked to nausea and vomiting [5], and to impairment of wellbeing [6] but it can be limited or prevented by insulin [7], preoperative infusion [8,9], or oral administration [10] of glucose. A nutritional carbohydrate drink given before surgery [11] might also prevent insulin resistance. However, whether the drink affects postoperative catabolism is unknown, although insulin resistance has been correlated with catabolism and intracellular dehydration after hysterectomy [12]. The benefit of a nutritional drink with regard to overall postoperative wellbeing is still uncertain [13].

The hypothesis of the present study was that some beneficial effects of a nutritional drink after elective hip replacement surgery could be attributed to the volume component of the drink. The research, therefore, assessed the relationship between fluid intake, with and without carbohydrates, on metabolism (glucose clearance, insulin sensitivity, stress, and muscle catabolism), body fluid volumes, surgical complications, and postoperative wellbeing. A recently validated glucose tolerance test assessed insulin sensitivity [14].

## Methods

### Patients

Between May 2008 and September 2009, 60 patients between 44 and 89 years of age (mean age, 69 years) undergoing elective total hip replacement under spinal anesthesia were studied in an open randomized clinical trial. Total hip replacement surgery implies that both the acetabular cup and the femoral head are replaced. The cup is usually made of plastic while the head is of metal. The components can be either cemented or just pressed firmly against the bone. Total hip replacement surgery was chosen for the study because the surgical procedure is well standardized and intake of a carbohydrate drink is not yet a routine, which is recommended before abdominal surgery [15].

Exclusion criteria were endocrinologic disorders, including diabetes, and treatment with cortisone. The Regional Ethics Committee of Stockholm (Ref. 2007/1628-31/4) approved the protocol. Each patient gave his/her informed consent to participate. The most common diseases affecting the patients included hypertension (*n* = 17), previous thrombosis (*n* = 4), chronic obstructive pulmonary disease (*n* = 3), and previous cardiovascular surgery (*n* = 3).

### Procedure

Each patient was randomly assigned to one of three study groups by the sealed envelope method after all baseline parameters had been measured in the orthopedics department on the day before the surgery. Before the study started, the surgeon (SL) prepared 60 blank envelopes in which the treatment group in the proportion 1:1:1 was written on a piece of blank paper. The research nurse drew the envelopes which were mixed together in a large bucket and opened each of them together with the patient. The groups were:

1. **Fasting**: no food or water from midnight before the surgery (control)

2. **Tap water**: 800 mL by mouth, 2 h before entering the operating room

3. **Nutrition**: a carbohydrate drink (50 kcal/100 mL; Preop^R^, NutriciaNordica AB, Stockholm, Sweden) 800 mL in the evening before the surgery (Day 0) and 400 mL 2 h before entering the operating room (Day 1)

Cemented total hip replacements were performed under spinal anesthesia (cement was avoided in three middle-aged patients). Patient monitoring consisted of pulse oximetry and non-invasive blood pressure and heart rate measurement. At the end of surgery, patients with more than minimal bleeding were given 1 g of tranexamic acid intravenously (Cyklokapron, Meda AB, Solna, Sweden).

Postoperative pain was managed using an epidural catheter with a continuous infusion of levobupivacaine and sufentanil (*n* = 22) or a wound catheter with ropivacaine and ketorolac (*n* = 25; the hospital practice changed during the study period). All patients were also given oral paracetamol. Oral oxycodone served as the rescue pain reliever.

### Physiological measurements

**Figure 1:**
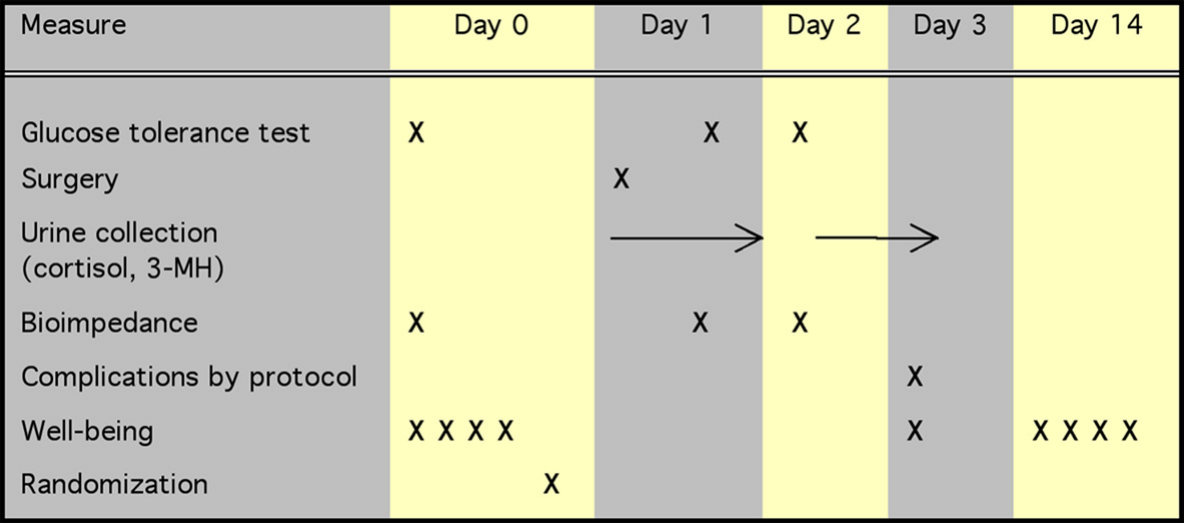
Flow chart showing the measurements taken in relation to the surgery.

Figure 1 shows the scheme used for these measurements.

#### Intravenous glucose tolerance test (IVGTT)

A short IVGTT was performed in the fasting state on Day 0, 2 to 3 h after surgery on Day 1, and before breakfast on Day 2. A bolus of 0.2 g/kg of glucose was injected as a 30% solution over 4 min, and the plasma glucose and insulin concentrations were measured at baseline and at 10, 20, 30, 45, 60, and 75 min. Plasma glucose was measured on a Modular P (Roche Diagnostics, Tokyo, Japan) and plasma insulin on a Roche-Hitachi Modular E170 (Hitachi, Tokyo, Japan).

#### Physical stress

The serum cortisol concentration was measured 2 to 3 h after the surgery ended; it was analyzed on the Roche-Hitachi Modular E170. The free cortisol fraction over time was estimated from the ratio of the cortisol and creatinine concentrations in the urine collected from a urinary bladder catheter inserted just before the operation started and up to the morning of Day 2. A second urinary collection began on the morning of Day 2 and ended on the morning of Day 3. The method used for the cortisol analysis was liquid chromatography tandem-mass spectrometry [16,17].

#### Muscle catabolism

The breakdown of muscular protein was quantified in the urine collected on Day 2 and Day 3 by measuring the ratio of 3-methylhistidine (3-MH) to creatinine, using a Biochrom 30 amino acid analyzer (Biochrom Ltd., Cambridge, UK) [18,19]. All patients were served lactovegetarian food from the day before surgery (Day 0) and throughout their hospital stay to prevent the confounding effects of exogenous meat on the excretion of 3-MH.

#### Body fluid volumes

Bioelectrical impedance analysis using a Xitron 4000B Spectrum Analyzer (Xitron Technologies Inc., San Diego, CA, USA) estimated the extracellular, intracellular, and total body fluid volumes (ECF, ICF, and TBW, respectively) [20,21]. Each value was the mean of three successive recordings based on the preoperative body weight.

#### Complications

Three approaches were used to collect data on complications. The hand-written medical records from the perioperative period and postoperative ward were used to calculate the mean systolic pressure and also to count the number of postoperative hypotensive events, defined as systolic arterial pressure <80 mmHg.

In the second approach, a research nurse used a checklist of 18 complications based on two published schemes [22,23] to register all postoperative adverse events up to Day 3. Finally, an orthopedic surgeon blinded to the randomization listed the complications during the entire hospital stay as noted by the hospital staff in the digital medical records.

### Calculations

#### Glucose kinetics

The kinetics of the glucose infused during the IVGTT was analyzed to characterize how effectively the study participants handled the exogenous glucose. In a one-compartment turnover model, the plasma concentration of glucose above baseline (*C*) at any time (t) resulting from the rate R_o_ was calculated from the following differential equation [12,14]:

(1) $$\frac{dC}{dt}=\frac{R_{o}}{V_{d}}-\frac{CL}{V_{d}}\cdot C(\text{t})$$

in which *V*_d_ is the volume of distribution and *CL* the clearance of glucose.

The best estimates and the associated standard deviations for the unknown parameters *V*_d_ and *CL* in this equation were estimated for each experiment individually by applying a non-linear least-squares regression routine, based on the Gauss-Newton method, to the analytical solution of the differential equation until no parameter changed by more than 0.1% in each iteration. Matlab version 4.2 (Math Works Inc., Natick, MA, USA) was used. The half-life (T_1/2_) was calculated as ln 2 ^.^*V*_d_ /*CL*.

#### Insulin sensitivity

The glucose kinetic parameters and the area under the curve indicating plasma insulin (AUC_ins_) above baseline during the IVGTT were used to estimate insulin sensitivity:

(2) $$M_{bw}=45 .4\cdot^{10}\log\left[\frac{CL\cdot 10^{6}}{V_{d}\cdot {AUC}_{ins}}\right]-2.5$$

The result was expressed as the M_bw_ of a hyperinsulinemic glucose clamp, to which the present approach was compared in non-diabetic volunteers with r = 0.80 and a 25th-75th prediction error of ±10% [14]. The same relationship has been previously evaluated as an index of insulin sensitivity from short IVGTTs in 496 diabetic and non-diabetic patients [24]*.*

Insulin sensitivity was also assessed with the ‘Quicki’ method [25], which uses the plasma glucose and insulin concentrations at baseline:

(3) $$\text{'Quicki'}=\frac{1}{{(}^{10}\text{log P}-\text{glucose}{+}^{10}\text{log P}-\text{insulin})}$$

In the equation, the symbols denote the 10-logarithm of the P-glucose (i.e. the plasma glucose) concentration.

### Wellbeing

Four scales previously used and validated in healthcare assessed life quality. The patient filled in these forms in the afternoon on the day before surgery. A research nurse interviewed the patients and filled in the forms once more 2 weeks after the surgery. The health index (HI) was also applied on the second postoperative day.

The wellbeing questionnaire (W-BQ12) consists of 12 items designed to acquire information about different aspects of psychological wellbeing [26,27]. The scale measures ‘energy’ and negative and positive wellbeing with four statements for each; the patient can respond using one of four levels. Higher values denote better general wellbeing.

Health index (HI) is a Swedish questionnaire in which the patient responds to 10 questions about ‘energy’, temper, mood, fatigue, loneliness, sleep, vertigo, bowel function, pain, mobility, and overall health during the past week [28]. When applied on the day after surgery, the questions pertain to the situation during the past 12 h. Answers can be given on four levels in which: 1 = very poor; 2 = rather poor; 3 = rather good; and 4 = very good. Hence, the highest total HI score for a patient is 40 and the lowest is 10. The test has been used to study self-perceived health status after ileal conduit urinary diversion [28] and after laparoscopic cholecystectomy [29].

Chalder’s Fatigue Scale (FQ) consists of 14 questions, which cover both physical and mental aspects of fatigue [30]. A ‘yes’ answer to a question about fatigue is graded as 1 and a ‘no’ answer as 2. Uncertain answers were graded as 1.5. The scale has been used to study fatigue in cancer [30] but also in other fields, such as in population surveys [31]. Translation from English to Swedish was checked through back-translation. The wording was virtually identical except in one question where ‘sleepy or drowsy’ was back-translated to ‘tired or lethargic’.

EQ-VAS is a ruler for rating self-perceived health; it is the last item of the EQ-5D [32].

### Statistics

The study was powered to detect a difference in the *CL* for glucose of 30% between any of the study groups and the control with a certainty of 90%. Reference data were obtained from both the preoperative and postoperative studies. In non-diabetic healthy humans *CL* has been 0.72 (mean, SD 0.18) [33] and 0.60 (0.26) [14]. During laparoscopy *CL* has been 0.21 (0.05) [34] and on the second postoperative day in hysterectomy patients 0.42 (0.08) [12]. The mean of these values yields a standardized difference of 1.05 which was entered into a nomogram showing that 18 patients per group were required [35]. The ability of the study to detect postoperative differences would be better than preoperative ones due to less expected scattering of those data.

The means (SD) of differences between the groups were evaluated with variance of analysis (ANOVA). When there was a skewed distribution, the data were expressed as the median (25th-75th percentile range), and the differences studied with the Kruskal-Wallis test. Changes during the study were evaluated with the Wilcoxon matched-pair test. Incidence data were studied with the chi-square test. Cronbach’s alpha coefficient was used to test the reliability of the tests of wellbeing. *P* <0.05 was considered statistically significant.

## Results

**Figure 2:**
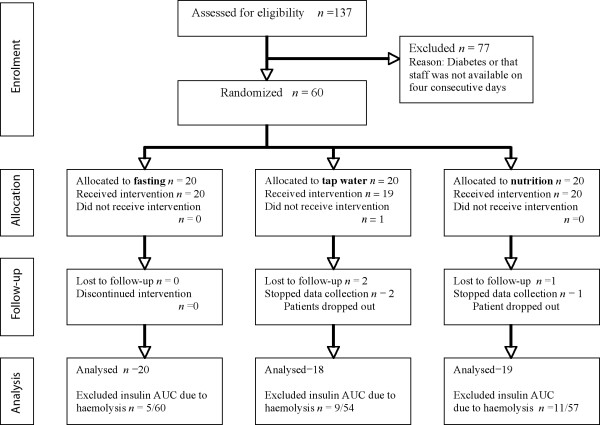
CONSORT diagram for the trial.

Sixty patients were randomly chosen and 57 completed the study (three dropped out due to personal reasons). Thirty of 171 insulin AUCs had to be discarded due to occasional hemolysis (Figure 2).

Data on the course of the surgery did not differ significantly between the study groups (Table 1). Overall, the operations lasted for an average of 106 (23) min and the average blood loss amounted to 400 (300–600) mL.

**Table 1:** Baseline characteristics of the patients and the operations

**Parameter**	**Fasting**	**Water**	**Nutrition**
	**(*n*** **= 20)**	**(*n*** **= 18)**	**(*n*** **= 19)**
Age (years)	68.5 (9.6)	66.4 (10.6)	65.2 (8.0)
Body weight (kg)	82.5 (20.0)	82.6 (10.8)	83.1 (13.8)
ASA class I/II/III (%)	16/63/21	6/69/25	24/63/13
Operating time (min)	115 (27)	103 (20)	99 (21)
Blood loss (mL)	450 (350–500)	400 (300–450)	400 (238–700)
Intravenous fluid (mL)			
Acetated Ringer’s	1000 (0)	1000 (0)	1000 (0)
Colloid fluid^a^	556 (162)	500 (177)	500 (177)
Epidural anesthesia after surgery (%)	35	33	47
Wound catheter after surgery (%)	40	56	37
Tranexamic acid (%)	50	56	26
Blood Hb			
Before surgery (g/dL)	13.32 (1.15)	13.53 (1.04)	13.56 (1.40)
Morning of Day 2 (g/dL)	10.33 (1.26)	9.72 (1.73)	10.41 (1.85)
Erythrocyte transfusion			
Frequency (%)	30	44	42
Units given (mean)	1.8	2.8	2.4
Body weight change Day 0 to Day 2 (%)^b^	+0.4 (2.3)	+1.9 (2.7)	+0.3 (1.7)

^a^Hydroxyethyl starch 130/0.4 (Voluven, Fresenius-Kabi).

^b^Only 12, 12, and 10 patients were weighed in each group on Day 3 as the others were unable to stand up on the scale.

Day 0, the day before surgery; Day 1, day of surgery; Day 2, first postoperative day; Day 3, second postoperative day.

### Glucose and insulin

The baseline concentrations of plasma glucose and insulin before surgery were virtually identical in the three study groups, the pooled data being 5.0 (0.6) mmol/L and 39 (26–71) pmol/L, respectively. Plasma glucose at baseline was 16% higher on Day 1 and a further 10% higher on Day 2 (both *P* <0.001). The participants’ baseline insulin was unchanged, except for an increase observed in the Nutrition group, on Day 2 (*P* <0.01).

#### Glucose kinetics

**Figure 3:**
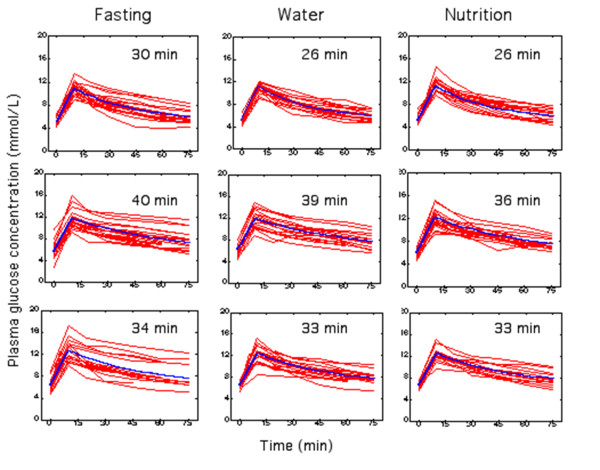
**The plasma glucose concentrations during the intravenous glucose tolerance tests (IVGTT).** Data are from Day 0 (top row), Day 1 (middle row), and Day 2 (bottom row) in the Fasting, Water, and Nutrition groups, respectively. Each thin line is one IVGTT test. The thick lines are the modeled curves based on the mean values for *V*_d_ and *CL* per each group and occasion.

**Figure 4:**
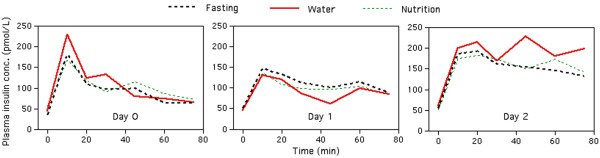
**The plasma insulin concentrations (median) during the IVGTT.** Data are from Day 0, Day 1, and Day 2 in the Fasting, Water, and Nutrition groups, respectively.

The mean plasma glucose during the IVGTTs on the three consecutive days was 8.3 (1.0), 9.7 (1.5), and 9.9 (1.3) mmol/L, respectively (Figure 3). Plasma insulin had decreased slightly in the postoperative ward (P <0.03), but then, rose by 18% on Day 2 (P <0.001; Figure 4).

*CL* was reduced by 23% (Day 1) and 11% (Day 2), with no significant differences between the groups. *T*_1/2_ increased by 60% (Day 1) and 35% (Day 2; Table 2).

**Table 2:** Changes in glucose kinetics and insulin sensitivity from before surgery up to the second postoperative day

**Parameter**	**Fasting**	**Water**	**Nutrition**
Glucose dose (g)	17.2 (3.8)	17.4 (2.5)	17.2 (3.0)
*Plasma glucose at baseline, change (%)*			
Day 0 to Day 1	+12 (22)	+20 (20)	+16 (17)
Day 0 to Day 2	+25 (15)	+28 (17)	+30 (13)
*Mean glucose during IVGTT (%)*			
Day 0 to Day 1	+14 (18)	+21 (18)	+19 (15)
Day 0 to Day 2	+21 (21)	+21 (16)	+21 (11)
***Glucose kinetics***			
*Volume of distribution, change (%)*			
Day 0 to Day 1	+10 (22)	+12 (25)	+7 (24)
Day 0 to Day 2	0 (4)	+5 (19)	+6 (22)
*Clearance, change (%)*			
Day 0 to Day 1	−23 (22)	−23 (28)	−23 (30)
Day 0 to Day 2	−14 (37)	−11 (37)	−9 (46)
*Half-life, T*_*1/2*_*, change (%)*			
Day 0 to Day 1	+57 (62)	+69 (84)	+59 (79)
Day 0 to Day 2	+34 (63)	+42 (74)	+35 (52)
***Insulin sensitivity***			
*Plasma insulin AUC above baseline (%)*			
Day 0 to Day 1^a^	−41 (−66 to −5)	−47 (−69 to −10)	−25 (−36 to +33)
Day 0 to Day 2^a^	+23 (−30 to +26)	+66 (+19 to +98)	+55 (+20 to +256)
*Insulin sensitivity change (%)*			
IVGTT Day 0 to Day 1^a^	−1 (−43 to +20)	+5 (−36 to +47)	−8 (−30 to +15)
Day 0 to Day 2^a^	−43 (−77 to +19)	−38 (−82 to −20)	−51 (−74 to 0)
‘Quicki’ Day 0 to Day 1	+2 (19)	+2 (8)	−5 (15)
Day 0 to Day 2	−5 (16)	−5 (14)	−15 (16)

^a^18% of the insulin AUC data are missing due to hemolysis.

Data are the mean (SD) or the median (25^th^-75^th^ percentile range).

Day 0, the day before surgery; Day 1, day of surgery; Day 2, first postoperative day; Day 3, second postoperative day.

#### Insulin

The mean plasma insulin concentration during the IVGTT decreased slightly in the postoperative ward (*P* <0.03), but then rose by 18% on Day 2 (*P* <0.001; Figure 4). The AUC changed by −36% (Day 1) and +51% (Day 2) as compared to the preoperative value (both *P* <0.001). The same pattern appeared in all three groups.

The insulin sensitivity on Day 0 (expressed as M_bw_) was 31 (19–41) μmol/kg/min when derived from the IVGTT. M_bw_ showed no statistically significant change from Day 0 to Day 1 but decreased on Day 2 (*P* <0.001). The decrease was much greater when based on the IVGTT (−48%) compared to ‘Quicki’ (−8%; Table 2).

### Physical stress, catabolism, and body fluid volumes

Plasma cortisol in the postoperative ward was 359 (182–601) nmol/L; no statistically significant differences were observed between the groups. Urine cortisol decreased by 26% between Day 1 and Day 2 (*P* <0.005; Table 3).

**Table 3:** Physical stress, catabolism, and changes in body fluid volume indices

**Parameter**	**Fasting**	**Water**	**Nutrition**
***Physical stress***			
Serum cortisol (nmol/L)			
Day 1	447 (161–620)	253 (118–544)	376 (222–553)
Urine cortisol/creatinine			
Day 1 to Day 2	40 (15–99)	41 (18–54)	41 (27–54)
Day 2 to Day 3	20 (7–38)	20 (8–33)	26 (14–34)
***Muscle catabolism***			
Urine methylhistidine/ creatinine			
Day 1 to Day 2	18.0 (2.0)	17.3 (2.6)	17.5 (2.7)
Day 2 to Day 3	19.3 (2.8)	18.1 (2.7)	18.6 (3.1)
***Body fluid volumes***			
ECF volume change (%)			
Day 0 to Day 2	−1 (4)	0 (6)	−2 (5)
Day 0 to Day 3	0 (6)	+1 (6)	−2 (6)
ICF volume change (%)			
Day 0-Day 2	+9 (22)	−2 (10)	+6 (11)
Day 0-Day 3	−1 (10)	+3 (23)	+8 (22)
TBW volume change (%)			
Day 0 to Day 2	+3 (8)	−1 (5)	+2 (5)
Day 0 to Day 3	−1 (5)	+2 (10)	+2 (9)

Data are the mean (SD) or the median (25^th^-75^th^ percentiles) depending on the distribution.

ECF, extracellular; ICF, intracellular; TBW, total body water.

Muscle catabolism, as indicated by the urinary 3-MH/creatinine ratio, did not differ significantly between the groups. The participants’ overall excretion decreased by 7% from Day 1 to Day 2 (*P* <0.015; Table 3).

The average ECF volume was unchanged by the surgery. The ICF volume increased by 4% on Day 1 and 3% on Day 2 (not significant, Table 3).

### Complications

No statistically significant differences were observed between the study groups with respect to peri- and postoperative hemodynamics.

The nurse’s follow-up showed that a mean of 1.5 complications occurred per patient by Day 3. Pain, nausea, and hypotension were most common (Table 4). Pain scores were virtually identical in those who received a local anesthetic via an epidural and a wound catheter (data not shown).

**Table 4:** Hemodynamic stability, complications, and length of hospital stay

**Parameter**	**Fasting**	**Water**	**Nutrition**
Systolic blood pressure, % of baseline (mean, SD)			
Baseline (mmHg)	155 (14)	149 (20)	158 (19)
Lowest during spinal anesthesia (%)	65 (19)	68 (12)	67 (13)
During surgery (%)	72 (17)	75 (10)	76 (7)
Postoperative ward (%)	70 (6)	68 (11)	66 (8)
Postoperative hypotension (%)^a^	30	50	32
Nurse*’*s follow-up^b^			
All ( mean per patient)	1.35	1.66	1.63
Pain	0.50	0.61	0.42
Nausea and vomiting	0.30	0.28	0.32
Hypotension	0.25	0.44	0.42
Food intolerance	0.10	0.11	0.11
0-1 complication (n)	12	9	10
≥ 2 complications (n)	8	9	9
All complications in digital journal (mean per patient)	0.30	0.39	0.21
Hospital stay (days)	6 (5–7)	6 (5–7)	5 (5–6)

^a^Drop in arterial systolic pressure to <80 mmHg.

^b^Erythrocyte transfusions are not included here but reported in Table 1.

Only a mean of 0.3 complications per patient was recorded in the digital journal.

The median hospital stay was one day shorter in the Nutrition group (5 days) than in the other two groups (6 days) but the difference was not statistically significant (P = 0.36).

### Wellbeing

**Figure 5:**
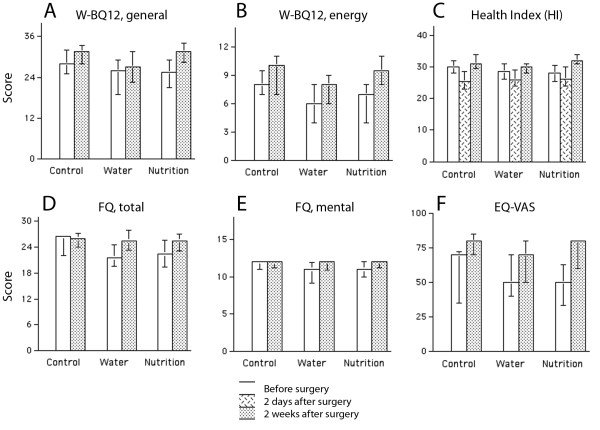
**Wellbeing and life quality shortly after elective total hip replacement surgery.** Bars = median values; Error bars = 25^th^ and 75^th^ percentiles.

W-BQ12 scores on general well-being increased after the surgery, from 27 (20–29) to 31 (25–33) points (*P* <0.0001) with no difference between the groups (Figure 5A, B).

The total HI score changed from 29 (24–32) before surgery to 26 (23–29; *P* <0.005) postoperatively, while it increased to 31 (29–34) 2 weeks later (*P* <0.001, Figure 5C). Two weeks postoperatively, HI was lower in the Water group compared to the Nutrition group (*P* <0.02).

The total FQ score changed from 23 (20–27) to 26 (24–27; *P* <0.001) with no difference between the groups (Figure 5 D, E).

The EQ-VAS score changed from 60 (40–70) before surgery to 75 (63–80) 2 weeks later (*P* <0.001) with no difference between the groups (Figure 5F).

The Cronbach’s alpha coefficients for the responses to the W-BQ12, HI, and FQ tests before surgery were 0.80 (mean of the three parts), 0.85, and 0.87, respectively. Two weeks after surgery, the corresponding values were lower: 0.72, 0.71, and 0.71.

## Discussion

Ingestion of a carbohydrate drink before surgery is believed to improve postoperative wellbeing by preventing the trauma-induced impairment of the glucose metabolism [10,11,36-38]. The present study was undertaken to examine whether these effects could at least partly be explained by the fact that the nutritional drink contains a large amount of water (1.2 L). This question was to be answered by studying if the results in the Water group would be close to those obtained in the Fasting group or in the Nutrition group. To our surprise, neither preoperative intake of tap water nor the nutritional drink did affect glucose kinetics, insulin sensitivity, hemodynamics, complications, hospital time, or wellbeing after elective hip replacement surgery. The study was powered to detect a difference in glucose clearance but some other parameters, such as insulin sensitivity and wellbeing, would probably need larger study groups to arrive at safe conclusions. However, the lack of even trends towards benefits for the interventions is striking.

There are reasons to believe that either tap water or nutrition should promote better-off patients. Hypovolemia is apparently common in the preoperative setting [39]; therefore, volume optimization should reduce the incidence of postoperative complications [40]. Those who received water were allowed 2 h to excrete excess fluid; doctors then assumed that the patients were well hydrated and that they were more likely to have a more normal blood volume when surgery started. However, no benefit from oral water over the fasting state was found.

Researchers have previously proposed that replacing preoperative fasting with caloric intake modified the metabolic response to surgery. The nutritional drink preparation, which loads the liver with carbohydrates, has been shown to preserve insulin sensitivity after colorectal [36] and hip surgery [37] but not after cardiac surgery [41]. Moreover, researchers have claimed that carbohydrate drinks improve wellbeing and reduce nausea and vomiting [8,9,38] although these results have not been consistent [13,42]; this study could not confirm the claim. Our results showed that preoperative fasting and the nutritional drink were followed by the same degree of urinary cortisol excretion, muscle catabolism, and length of hospital stay.

The present study adds to our knowledge about the time course of the development of postoperative insulin resistance. Hyperglycemia developed within hours after the surgery, but it was matched by the inhibition of the insulin response to glucose. Insulin resistance did not develop until the next day, and was then associated with a boosted insulin response to glucose. This sequence of events agrees with that of adolescents who develop type 2 diabetes [43]. Another observation is that high cortisol excretion preceded an increase in muscle catabolism by 1 day.

Insulin sensitivity was quantified with an IVGTT [14,24], which, in contrast to the hyperinsulinemic glucose clamp, provides information about the intrinsic insulin response. The IVGTT induces a smaller insult to normal physiology than the clamp, and therefore, lends itself to repetition during the postoperative follow-up.

Insulin sensitivity was also assessed with the ‘Quicki’ equation, which, just like the older HOMA test, uses the baseline values of plasma glucose and insulin. These simple tests are intended for population studies but have also been applied after surgery [38,41]. The baseline values were much less affected by the surgery (−8%) compared to the glucose tolerance test (−48%), which provided results more in accordance with those of the hyperinsulinemic clamp [10,37].

The scales describing perceived health and wellbeing yielded higher values than before the operation as little as 2 weeks after hip replacement surgery. The difference ranged between 7% and 30%. The W-BQ12 and FQ scales support that the improvements can be attributed to several aspects of wellbeing, such as perceived ‘energy’ and both mental and physical health. As expected, a dip in the HI score occurred 2 days after the surgery, but for ethical reasons, not all protocols for evaluating wellbeing were used in the early postoperative period.

The scales selected for the study ask questions pertinent to early convalescence. Several scales were used to obtain as complete view of the postoperative wellbeing as possible. Previous studies of health after total hip replacement have focused on the outcome of the implant, and therefore, used a follow-up period of 3 to 12 months [44,45]. They confirmed that the surgery improved both physical and mental aspects of wellbeing [46]. EQ-VAS applied before, and 1 year after the surgery, yielded almost identical values as those reported here after only 2 weeks. This suggests that most or all of the improvement in wellbeing occurs early after the hip replacement surgery.

The capacity of the preoperative nutritional drink to preserve insulin sensitivity is a key argument for recommending its use before surgery [15]. This evidence stems from two small studies. Insulin sensitivity was better maintained in six patients who were given the carbohydrate drink before colorectal operations compared to seven patients who were in the fasting state [36]. The conclusion is valid only by assuming a linear relationship between insulin resistance and the operating time, which was not found in the present study (data not shown).

The same carbohydrate drink preserved insulin sensitivity to a higher degree in eight patients than in another seven who received the placebo drink before elective hip surgery. However, the latter group was younger and had 22% higher insulin sensitivity at baseline [37]. A third reference on this topic provides a duplicate report summarizing the findings of the first two [10]. Other authors have investigated this issue by using the HOMA test; these investigations both support [38] and refute [41] the hypothesis.

The present study has several limitations. The trial was non-blinded as blinding was considered difficult to carry out in the fasting group. The difference in volumes of fluid administered 2 h before the surgery was due to the differences in volumes of distribution between tap water (TBW) and a carbohydrate drink (ECF volume) [12]. The Fasting group received 10% more colloid fluid during the surgery than the other two groups, but they also bled 13% more. The study layout was based on the assumption that the nutritional drink would preserve insulin sensitivity and reduce the incidence of complications, whereby focus was on postoperative comparisons between the groups. This fact explains why cortisol and 3-MH were not measured preoperatively..

Another limitation was that insulin sensitivity was not measured with the clamp technique, which is the golden standard method, although intravenous glucose tolerance is a widely accepted alternative [47]. The relatively sparse sampling scheme, motivated by ethical considerations of maximum blood sampling volume, has recently been validated against the hyperglycemic glucose clamp [14,24]. However, hemolysis in some insulin samples made the reported values too uncertain to be included in calculations of the AUC. Therefore, 18% of the AUCs for insulin had to be deleted, which resulted in insulin sensitivity being based on fewer data than originally intended.

## Conclusions

This randomized controlled trial shows that neither the preoperative intake of tap water nor a nutritional drink alleviates the reduction of the glucose clearance that occurs after elective hip replacement surgery. Moreover, there was no evidence that these treatments affected insulin sensitivity, hemodynamics, the incidence of postoperative complications, or wellbeing.

## Abbreviations

AUC, Area under the curve; CL, Clearance; ECF and ICF, Extra- and intracellular fluid; HOMA, Homeostatic model assessment; IVGTT, Intravenous glucose tolerance test; ‘Quicki’, Quantitative insulin sensitivity check index; TBW, Total body water; Vd, Volume of distribution; W-BQ12, A well-being questionnaire with 12 questions; 3-MH, 3-methyl-histidine.

## Competing interests

The authors declare that they have no competing interests.

## Authors’ contributions

SL recruited and informed the patients, was responsible for references, and co-wrote the manuscript. RH planned the study, wrote appropriate applications, and had the main responsibility for the content of the manuscript. Both authors read and approved the final manuscript.

## Authors’ information

Stefan Ljunggren is MD and specialist in orthopedics. He is currently a PhD Student. Robert G Hahn is MD is professor of anesthesiology and intensive care.
